# A translational multimodal machine‐learning prototype predicting valproate response in epilepsy treatment

**DOI:** 10.1002/epi.70300

**Published:** 2026-05-20

**Authors:** Simeon Platte, Afsheen Kumar, Giorgia Guerini, Massimo Pandolfo, Denise Haslinger, Colin B. Josephson, Guillermo Delgado‐García, Navprabhjot Kaur, Michaela‐Pauline Lux, Heiko Stempfle, Chantal Depondt, Reetta Kälviäinen, Felix Rosenow, Sophie von Brauchitsch, Karl Martin Klein, Andreas G. Chiocchetti

**Affiliations:** ^1^ Department of Child and Adolescent Psychiatry, Psychosomatics, and Psychotherapy Goethe University Frankfurt, University Hospital Frankfurt Germany; ^2^ Laboratory of Experimental Neurology Université Libre de Bruxelles Brussels Belgium; ^3^ Department of Neurology and Neurosurgery, Montreal Neurological Institute McGill University Montreal Quebec Canada; ^4^ Department of Clinical Neurosciences, Cumming School of Medicine University of Calgary Calgary Alberta Canada; ^5^ Hotchkiss Brain Institute University of Calgary Calgary Alberta Canada; ^6^ O'Brien Institute for Public Health, Cumming School of Medicine University of Calgary Calgary Alberta Canada; ^7^ Centre for Health Informatics, Cumming School of Medicine University of Calgary Calgary Alberta Canada; ^8^ Institute of Health Informatics University College London London UK; ^9^ Social Work in Epilepsy Association Kehl‐Kork Germany; ^10^ German Epilepsy Association Berlin Germany; ^11^ Department of Neurology, Erasmus Hospital, Hôpital Universitaire de Bruxelles Université Libre de Bruxelles Brussels Belgium; ^12^ Kuopio Epilepsy Center, Neurocenter Kuopio University Hospital, member of European Reference Network for Rare and Complex Epilepsies EpiCARE Kuopio Finland; ^13^ Institute of Clinical Medicine, School of Medicine, Faculty of Health Sciences University of Eastern Finland Kuopio Finland; ^14^ Department of Neurology Goethe University Frankfurt, University Hospital, Epilepsy Center Frankfurt Rhine‐Main Frankfurt Germany; ^15^ Goethe University Frankfurt, University Hospital, LOEWE Center for Personalized Translational Epilepsy Research Frankfurt Germany; ^16^ Department of Medical Genetics, Cumming School of Medicine University of Calgary Calgary Alberta Canada; ^17^ Department of Community Health Sciences University of Calgary Calgary Alberta Canada; ^18^ Alberta Children's Hospital Research Institute University of Calgary Calgary Alberta Canada

**Keywords:** antiseizure medications, biomarker‐based machine learning classifier, personalized treatment

## Abstract

**Objective:**

Epilepsy affects ~1% of the global population and often requires lifelong antiseizure medication (ASM) therapy. Valproic acid (VPA) is a commonly prescribed first‐line ASM, yet only approximately half of patients achieve sustained seizure freedom. Treatment selection remains largely empirical. We aimed to develop and independently validate a multimodal predictive model to estimate response to VPA and support more individualized treatment strategies.

**Methods:**

This cross‐sectional treatment response modeling study used data from a subset of the international Epi25 cohort (Belgium, Finland, Germany). Individuals with epilepsy were included if they had received VPA monotherapy and had available genetic or clinical data. Discovery data (1965–2021, 58% female) were split into a training set (*n* = 196) and test set (*n* = 133). Independent validation was performed in a Canadian cohort (2021–2022, *n* = 156, 40% female). The primary outcome was binary VPA response. Responders achieved ≥12 months of seizure freedom attributed to VPA; nonresponders had >50% seizure recurrence or discontinued VPA due to inefficacy, adverse effects, or unclear reasons. The predictive algorithm integrated features derived from common and rare variants in VPA pharmacokinetic and pharmacodynamic genes, in vitro neuronal VPA response measures, and clinical features. Model performance was assessed using accuracy, predictive values (negative predictive value [NPV]/positive predictive value [PPV]), and area under the curve (AUC).

**Results:**

In the independent validation cohort, the multimodal classifier achieved a balanced accuracy of 63% (95% confidence interval [CI] = 52%–73%), NPV of 70% (95% CI = 51%–85%), PPV of 60% (95% CI = 46%–72%), and AUC of .73 (95% CI = .63–.83). Models restricted to single or dual data modalities showed consistently lower predictive performance.

**Significance:**

This proof‐of‐concept study demonstrates that integrating genetic, cellular, and clinical data enables prediction of VPA treatment response with clinically meaningful accuracy. Although not yet ready for clinical application, this approach supports the feasibility of biomarker‐informed ASM selection and may ultimately reduce time to effective seizure control.

**Key points**

- Multimodal integration of genetic, cellular, and clinical data enables prediction of VPA treatment response.
- The model achieved 63% balanced accuracy and .73 AUC in an independent validation cohort.
- Multimodal models outperformed single‐modality approaches, exceeding clinical predictors alone.
- Early identification of VPA responders could reduce trial‐and‐error delays by ~60% in modeled estimates.
- This proof‐of‐concept supports biomarker‐informed antiseizure medication selection in epilepsy.

## 1 INTRODUCTION

Epilepsy affects ~1% of the population. It is characterized by recurrent seizures and usually requires long‐term antiseizure medication (ASM).^1^ The current strategy for identifying the best treatment is based on clinically guided trial‐and‐error. Only ~50% of patients remain seizure‐free for at least 12 months with first‐line ASM, 12% with the second, and 4% with the third^2^, ^3^, ^4^; 33% of patients exhibit incomplete response to ASMs.^3^, ^5^ The median time to failure is 90 days due to unacceptable adverse reactions (UARs) and 234 days due to inadequate seizure control (ISC).^6^ An informed treatment decision could substantially reduce the time required to achieve seizure freedom, improve quality of life and medication adherence,^7^ and decrease the staffing requirements and thus overall costs.^3^

Although many new ASMs have been introduced in the past years, there has been little improvement in overall treatment success rates.^4^, ^6^ This underpins the need for personalized or subgroup‐specific treatments, rather than a one‐size‐fits‐all approach.^7^ One of the most prescribed first‐line ASMs, since its approval in 1967, is valproic acid (VPA) in children and adolescents (29.6%) and adults (26.5%).^8^ Its use in females has declined due to its teratogenic effects.^9^

However, 40%–50% of patients experience ISC or UARs and discontinue the medication (37%).^4^, ^6^ The main VPA UARs include hepatotoxicity, pancreatitis, weight gain, irregular periods, cognitive difficulties (e.g., spatial memory), alopecia, tremors, osteoporosis, and teratogenicity. Several mechanisms have been hypothesized to be associated with UARs of VPA^10^ such as mitochondrial dysfunction, carnitine shortage, immune‐mediated reactions, glutathione depletion, or leptin and insulin resistance.^11^ However, the pathophysiologically relevant genes and variations are not as well defined as VPA's pharmacokinetic and pharmacodynamic pathways.

Here, we developed a predictive algorithm to support treatment decision for VPA in epilepsy including genetic variation in genes associated with (1) VPA pharmacodynamics and pharmacokinetics as well as (2) cellular response to VPA. VPA was selected due to its high representation in historical cohorts enabling adequately powered analyses, its comparatively well‐characterized molecular mechanisms, and its continued clinical relevance despite current prescribing restrictions.

VPA is metabolized via glucuronidation, β‐oxidation in the mitochondria, and to a lesser extent cytochrome P450‐mediated oxidation.^12^ At the functional level, VPA has been identified to act on γ‐aminobutyric acid (GABA) levels in the brain by blocking the major anabolic enzymes 4‐aminobutyrate aminotransferase, aldehyde dehydrogenase 5 family member A1, and oxoglutarate dehydrogenase.^13^ VPA further blocks voltage‐gated ion channels CACNA1C, CACNA1D, CACNA1N, and CACNA1F and the SCN family. In addition, it has been reported to bind the histone acetylases HDAC1 and HDAC9,^14^, ^15^ increasing the expression of antiapoptotic and antitumor genes. We extended this list of candidate genes including those regulated upon response to VPA in human GABAergic and glutamatergic neurons, as assessed in vitro.

As part of the EU‐funded RAISE‐GENIC consortium, we developed a multimodal machine learning pipeline predicting VPA response including data on individuals' clinical profiles and the genetic variants of genes associated with VPA metabolism, function, and gene–network regulation and tested whether we can predict the response to treatment. This prototype has been developed together with patient representatives and was trained and tested in a multiethnic cohort and validated in an independently collected sample.

## 2 MATERIALS AND METHODS

**FIGURE 1:**
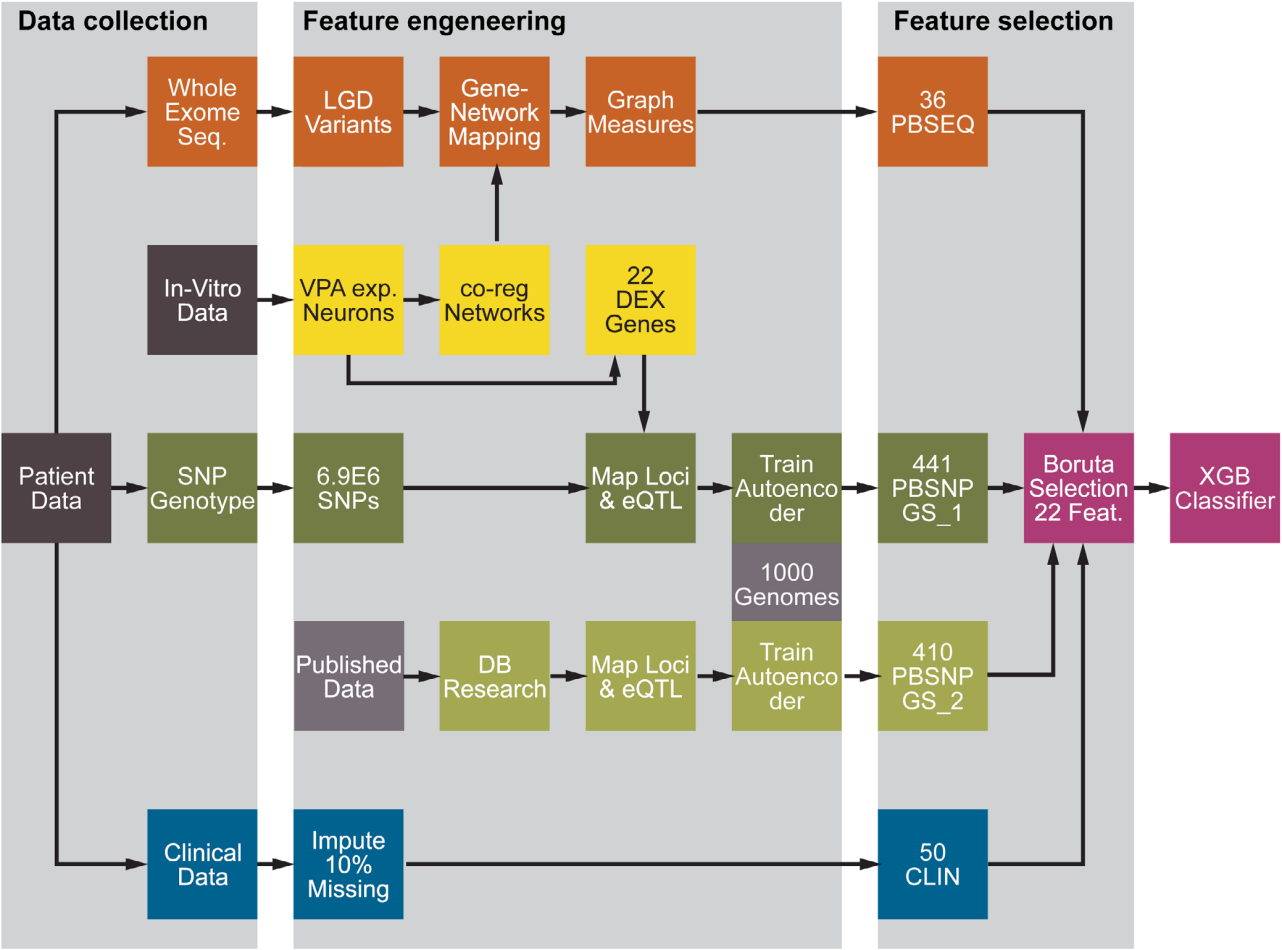
Schematic overview of the implemented multimodal pipeline. The final Classifier (purple) integrated three different feature sets: network‐based features derived from whole exome sequencing (PBSEQ) and common variation derived features of valproic acid (VPA)‐associated pathways (PBSNP) from two gene sets (GS_1, GS_2). Newly generated data (dark gray) and published data (light gray) were included to define these gene sets. The pipeline was implemented and trained on a train–discovery cohort. Parameters and models were then tested in the test–discovery cohort and finally validated in an independent validation cohort. CLIN, clinical features; co‐reg, co‐regulated; DB, database; DEX, differentially expressed; eQTL, expression quantitative trait loci; exp., exposed; Feat., features; LGD, likely gene‐disrupting (variants); PBSEQ, pathway‐based sequencing; PBSNP, pathway‐based SNPs; Seq., sequencing; SNP, single nucleotide polymorphism; XGB Classifier, EXtreme Gradient Boosting Classifier (XGBoost).

We built a multimodal pipeline (Figure 1) to predict VPA response. The model was trained and tested on a discovery cohort (*n* = 329, Epi25) and validated on an independent cohort (*n* = 202). Full methods are provided in Supplementary Material S1.

### 2.1 Definition of gene sets

#### 2.1.1 Gene Set 1: VPA‐associated genes and gene networks

Human induced pluripotent stem cells (iPSCs) derived from two healthy donors were differentiated to cortical progenitors and neurons for 40 days as published.^16^ On day 35 (D35), cells were exposed to .0625–1.0 mmol·L^−1^ VPA,^17^ (Figure S1). On D40, RNA was extracted and 3′ RNA sequenced (single end, two replicates per condition; *n* = 12), detecting 15 629 genes after quality control (QC); all samples passed QC (Figure S2). Differential gene expression analysis was performed using linear mixed models (DESeq2),^18^ as published.^19^ Differentially expressed genes (DEX) replicated in both cell lines (false discovery rate [FDR] < .05, |log_2_FC| > 1) formed Gene Set 1. To identify VPA‐responsive gene modules, we applied weighted gene coexpression network analysis (WGCNA).^20^ DEX modules replicated with FDR < .05 and |log_2_FC| > 1 were included.

#### 2.1.2 Gene Set 2: Pharmacogenomics pathways

Gene Set 2 comprised 12 VPA metabolism‐associated genes (cytochrome P450 and UDP‐glucuronosyltransferases) retrieved from DrugBank (ID: DB00313, accessed September 14, 2023; Table S1).

### 2.2 Data description

#### 2.2.1 Pathway‐based single nucleotide polymorphisms embedding cohort

Single nucleotide polymorphism (SNP)‐based autoencoders (AEs) were trained on 3157 samples from the 1000 Genomes Phase 3 dataset,^21^ filtered to 4.1Mio SNPs shared with the discovery cohort.

#### 2.2.2 Discovery cohort

SNP data from 1334 epilepsy patients across three Epi25^22^, ^23^ sites (Brussels *n* = 387, Frankfurt *n* = 259, and Kuopio *n* = 688) were included. After QC and imputation as published,^24^ 1327 individuals with 4.1 million high‐quality SNPs remained. Whole exome sequencing (WES) was available for 1319 individuals after QC; likely gene‐disrupting (LGD) variants were identified using ANNOVAR.^25^ Clinical data were available for 1248 patients, including seizure/epilepsy type (Table S2). After excluding four individuals with >10% missing clinical data, remaining gaps were imputed (MICE^26^ or zero‐imputation of sparse binary features).

#### 2.2.3 Treatment response

Of those individuals who had at least one feature modality present, 417 received VPA as monotherapy at any given timepoint. Treatment outcomes were manually classified following EpiPGX consortium definitions. Responders achieved ≥12 months of seizure freedom attributed to VPA. Nonresponders had seizure recurrence at >50% of the pretreatment frequency after VPA had been adequately applied. If individuals had valid treatment data available but met neither the definition of response nor of nonresponse, they remained unclassified. This included partial response with seizure recurrence up to 50% and discontinuation of VPA due to adverse effects, before response or nonresponse could be assessed. A total of 88 cases had no information on treatment response documented and were excluded. Following clinical and patient advisory input, unclassified individuals were grouped with nonresponders to reflect overall treatment success in clinical practice including tolerability. Hence, for the analysis the following treatment outcomes were compared: (1) responders: patients who achieved ≥12 months of seizure freedom attributed to VPA (*n* = 156); and (2) nonresponders: patients with ongoing seizures on VPA or patients who stopped VPA (*n* = 173).

#### 2.2.4 Independent validation

The independently recruited cohort from Calgary included 183 patients with epilepsy with VPA treatment after exclusion of unknown responders. Preprocessing was performed for all data modalities as above. After QC, SNP data were available for 153 individuals with >94% coverage between cohorts (missing data were imputed using major alleles); WES data were available for 155 individuals; and clinical variables were available for 136 patients. A total of 156 patients had at least one feature modality available (49 responders, 134 nonresponders).

### 2.3 Feature engineering

#### 2.3.1 PBSNP feature engineering

To compress genetic variation, one undercomplete AE per gene set was trained on the 1000 Genomes dataset (80/20 train–test split). SNPs within ±2 kb of genes plus GTEx v8 brain expression quantitative trait loci (eQTLs)^27^ were included. Variants in linkage disequilibrium (*R* ^2^ > .8) were pruned, yielding 4093 SNPs (Gene Set 1) and 27 870 SNPs (Gene Set 2), respectively. Minor allele dosages were scaled to 0, .5, and 1. AEs used the Adam optimizer, LeakyReLU activation, and mean squared error (MSE) loss. Bottleneck size was set to 10% of input size; batch size was 32. A grid search (36 models; Table S3) varied dropout rates, activation functions, and network depth. Models were trained for up to 50 epochs with early stopping and evaluated via threefold cross‐validation. Final models were retrained for up to 500 epochs and validated on the 20% holdout set. Bottleneck layer outputs from the best models were used as compressed PBSNP features for each patient.

#### 2.3.2 Pathway‐based sequencing features: Network‐based features from exome data

Pathway‐based sequencing (PBSEQ) features captured graph‐based properties of the VPA‐responsive gene modules. For each module, 12 graph metrics were computed from the gene–gene correlation matrix, including weighted (e.g., mean/sum connectivity, path length, skewness) and unweighted (e.g., density, betweenness, transitivity) parameters. For unweighted measures, edge correlations were thresholded preserving 95% of node connectivity. To calculate patient‐specific features, genes with LGD variants were removed from the graph, and network metrics were recalculated.

### 2.4 Multimodal data integration

#### 2.4.1 Discovery cohort

Features from three datasets (i.e., PBSNP [Gene Sets 1 & 2], PBSEQ, and CLIN [clinical data]) were combined for 329 patients with VPA outcome data.

#### 2.4.2 Validation cohort

Integration followed the same approach, yielding 156 individuals with complete multimodal data.

### 2.5 Classifier/recommender model

The discovery cohort was split into a training set (60%) and holdout test set (40%), preserving responder ratios (47%). SMOTE (Synthetic Minority Oversampling Technique)^28^ was applied independently to each dataset to balance classes, resulting in ~5% synthetic responders per set. Features were minimum–maximum scaled on the training set and applied to test and validation sets. All subsequent processing and model training were conducted exclusively on the training set, with fixed parameters and models subsequently applied unchanged to the test and validation sets.

#### 2.5.1 Feature selection

Features were selected using the Boruta^29^ algorithm (*p* > .01, maximum = 200 runs). The union of features from 10 runs was used to maximize relevant predictors.

#### 2.5.2 Model training

A gradient‐boosted decision tree classifier (XGBoost)^30^ was trained on selected features to predict response. Hyperparameters were tuned via cross‐validated grid search (early stopping = 20, maximum = 1000 rounds; Table S4). The best model (binary loss entropy) was retrained (early stopping = 30, maximum = 5000 rounds).

#### 2.5.3 Validation

Model performance was assessed on the holdout test set and validated on the additionally recruited independent validation cohort using the full pipeline. Model performance metrics were calculated with 95% confidence intervals (CIs) using DeLong's method for area under the curve (AUC) and exact binomial (Clopper–Pearson) methods where appropriate.

#### 2.5.4 Model comparison

To assess modality contribution, six additional models using individual or paired feature types (PBSNP, PBSEQ, CLIN) were trained and validated using the same full pipeline. Model performances were compared pairwise with the full multimodal model using DeLong test for correlated receiver operating characteristic curves, with differences in classification accuracy assessed by McNemar test on paired predictions.

### 2.6 Avoidable delay estimation

The avoidable delay relates to the partition of patients who do not respond to the first ASM, but to second or third. Each proportion is weighted by the empirical median days to failure for UARs or ISC (50/50 mean) as published.^6^ We then assumed (1) an increased first‐line response rate (yielding 59% responders with the final classifier), (2) a fixed overall balance of responders versus drug‐resistant cases across three successive ASM regimens, and (3) proportional downscaling of response rates for the second and third regimens. The mean avoidable delay was then calculated relative to the as‐is scenario (i.e., 50% responders at first ASM). Estimations were restricted to ASM regimens 1–3, as additional regimens yielded ≤1% incremental response. The estimates thus rely on published response rates across treatment lines from observational data,^3^ which do not necessarily transfer to the scenario where all received VPA first‐line. To contextualize an upper bound under randomized conditions, results from the randomized controlled trial of the Standard and New Antiepileptic Drugs Study (SANAD RCT; Arm B, VPA as standard initial therapy) suggest ~53% first‐line VPA success (approximation based on retention rate and 12‐month seizure freedom).^6^ This is broadly consistent with prior observational estimates but underscores uncertainty in the avoidable‐delay calculation. Full assumptions are detailed in Table S8.

### 2.7 Ethics and patient consent statements

Informed consent for the EPi25 Collaborative has been documented.^22^ The recruitment of the validation cohort in Canada was approved by the Conjoint Health Research Ethics Board at the University of Calgary (REB18‐2099). Collection and generation of iPSCs was approved by the institutional ethics committee of Erasmus Hospital, Brussels (P2008.313). All participating individuals provided written informed consent.

## 3 RESULTS

### 3.1 Sample characteristics

The overall discovery cohort included 329 individuals with genetic and clinical data available. As expected, nonresponders showed significantly higher rates of drug resistance (variable not included in classifiers), focal epileptiform discharges, focal seizures, and bilateral seizures (odds ratio > 1) and lower rates of photoparoxysmal reactions, generalized seizures, and myoclonic seizures (Table 1). No significant differences were found for age at onset, age at study enrolment, or sex.

**TABLE 1:** Descriptive statistics: Discovery cohort.

Characteristic	Responders, *n* = 156	Nonresponders, *n* = 173	OR [95% CI]	*p* ^a^
Cohort				
Test–discovery	63 (40.4%)	70 (40.5%)		
Train–discovery	93 (59.6%)	103 (59.5%)	1.00 [.64–1.55]	1.000
Sex				
Female	91 (58.3%)	100 (57.8%)		
Male	65 (41.7%)	73 (42.2%)	1.02 [.66–1.59]	1.000
Enrollment age	44.1 (17.9)	47.8 (17.7)		.062
Age at onset	21.6 (17.5)	20.6 (16.1)		.583
Ethnicity PC1	.020 (.018)	.016 (.023)		.053
Ethnicity PC2	−.004 (.011)	−.005 (.033)		.780
Ethnicity PC3	.001 (.011)	−.003 (.046)		.212
Ethnicity PC4	−.001 (.011)	.001 (.033)		.440
Autism	0 (0%)	0 (0%)		
Intellectual disability	10 (6.4%)	12 (6.9%)	1.09 [.45–2.67]	1.000
Psychosis	6 (3.8%)	10 (5.8%)	1.52 [.54–4.64]	.577
Epileptic spasms	0 (0%)	0 (0%)		
Focal epileptiform discharges	49 (31.4%)	88 (50.9%)	2.25 [1.44–3.56]	.001
Photoparoxysmal reactions	5 (3.2%)	0 (0%)	Inf	.023
Unspec. epil. abnorm.	8 (5.1%)	12 (6.9%)	1.37 [.54–3.63]	.650
Focal seizures	90 (57.7%)	149 (87.1%)	4.92 [2.88–8.69]	<.001
Febrile seizures	15 (9.6%)	24 (13.9%)	1.51 [.76–3.06]	.307
Bilateral tonic–clonic seizures	70 (45.2%)	106 (61.3%)	1.92 [1.24–2.99]	.005
Generalized seizures	48 (31.0%)	11 (6.4%)	.15 [.07–.30]	<.001
Tonic seizures	0 (0%)	1 (.6%)	Inf	1.000
Atonic seizures	3 (1.9%)	2 (1.2%)	.61 [.07–4.04]	.670
Myoclonic seizures	24 (15.4%)	8 (4.7%)	.27 [.11–.61]	.002
Absence seizures	24 (15.4%)	5 (2.9%)	.17 [.05–.42]	<.001
Atypical absence seizures	2 (1.3%)	2 (1.2%)	.88 [.09–8.55]	1.000
Other seizures	1 (.6%)	2 (1.2%)	1.73 [.14–54.8]	1.000

Abbreviations: CI, confidence interval; Inf, infinity; OR, odds ratio; PC, principal component; Unspec. epil. abnorm., unspecified epileptiform abnormalities.

^a^ Uncorrected test of significance: χ^2^ for dichotomous and *t*‐test for continuous variables.

After stratified randomization, the only difference between the training and test sets was a higher proportion of females in the training set (*p* = .041; Table S5).

The validation cohort showed a similar clinical profile to the discovery cohort (Table 2). Nonresponders had higher rates of focal discharges and focal and bilateral seizures and lower rates of photoparoxysmal reactions and generalized and myoclonic seizures as well as a higher prevalence of intellectual disability. No differences were observed in sex, age at onset, or age at study.

**TABLE 2:** Descriptive statistics: Validation cohort.

Characteristic	Responders, *n* = 46	Nonresponders, *n* = 110	OR [95% CI]	*p* ^a^
Sex				
Female	24 (52.2%)	39 (35.5%)		
Male	22 (47.8%)	71 (64.5%)	1.98 [.98–4.01]	.057
Enrollment age	32.2 (13.463)	34.9 (14.350)		.288
Age at onset	13.8 (11.455)	14.6 (14.028)		.719
Ethnicity PC1	.009 (.033)	−.001 (.049)		.140
Ethnicity PC2	<.001 (.022)	.004 (.047)		.588
Ethnicity PC3	−.002 (.006)	.010 (.077)		.117
Ethnicity PC4	.002 (.017)	−.004 (.091)		.493
Autism	1 (2.4%)	5 (5.3%)	2.00 [.29–54.1]	.668
Intellectual disability	5 (12.2%)	32 (33.7%)	3.55 [1.35–11.3]	.018
Psychosis	0 (0%)	2 (2.1%)	Inf	1.000
Spasms seizures	0 (0%)	1 (1.1%)	Inf	1.000
Focal epileptiform discharges	10 (24.4%)	64 (67.4%)	6.25 [2.79–15.1]	<.001
Photoparoxysmal reactions	5 (12.2%)	1 (1.1%)	.09 [.00–.59]	.010
Unspec. epil. abnorm.	0 (0%)	1 (1.1%)	Inf	1.000
Focal seizures	9 (22.0%)	67 (70.5%)	8.27 [3.60–20.7]	<.001
Febrile seizures	3 (7.3%)	10 (10.5%)	1.44 [.40–7.04]	.754
Bilateral seizures	11 (26.8%)	61 (64.2%)	4.80 [2.18–11.2]	<.001
Generalized seizures	24 (58.5%)	21 (22.1%)	.20 [.09–.45]	<.001
Tonic seizures	1 (2.4%)	5 (5.3%)	2.00 [.29–54.1]	.668
Atonic seizures	0 (0%)	8 (8.4%)	Inf	.105
Myoclonic seizures	13 (31.7%)	11 (11.6%)	.29 [.11–.71]	.010
Absence seizures	19 (46.3%)	12 (12.6%)	.17 [.07–.40]	<.001
Atypical absence seizures	0 (0%)	1 (1.1%)	Inf	1.000
Other seizures	0 (0%)	2 (2.1%)	Inf	1.000

Abbreviations: CI, confidence interval; Inf, infinity; OR, odds ratio; PC, principal component; Unspec. epil. abnorm., unspecified epileptiform abnormalities.

^a^ Uncorrected test of significance: χ^2^ for dichotomous and *t*‐test for continuous variables.

### 3.2 iPSC results and definition of Gene Set 1 and gene networks

**FIGURE 2:**
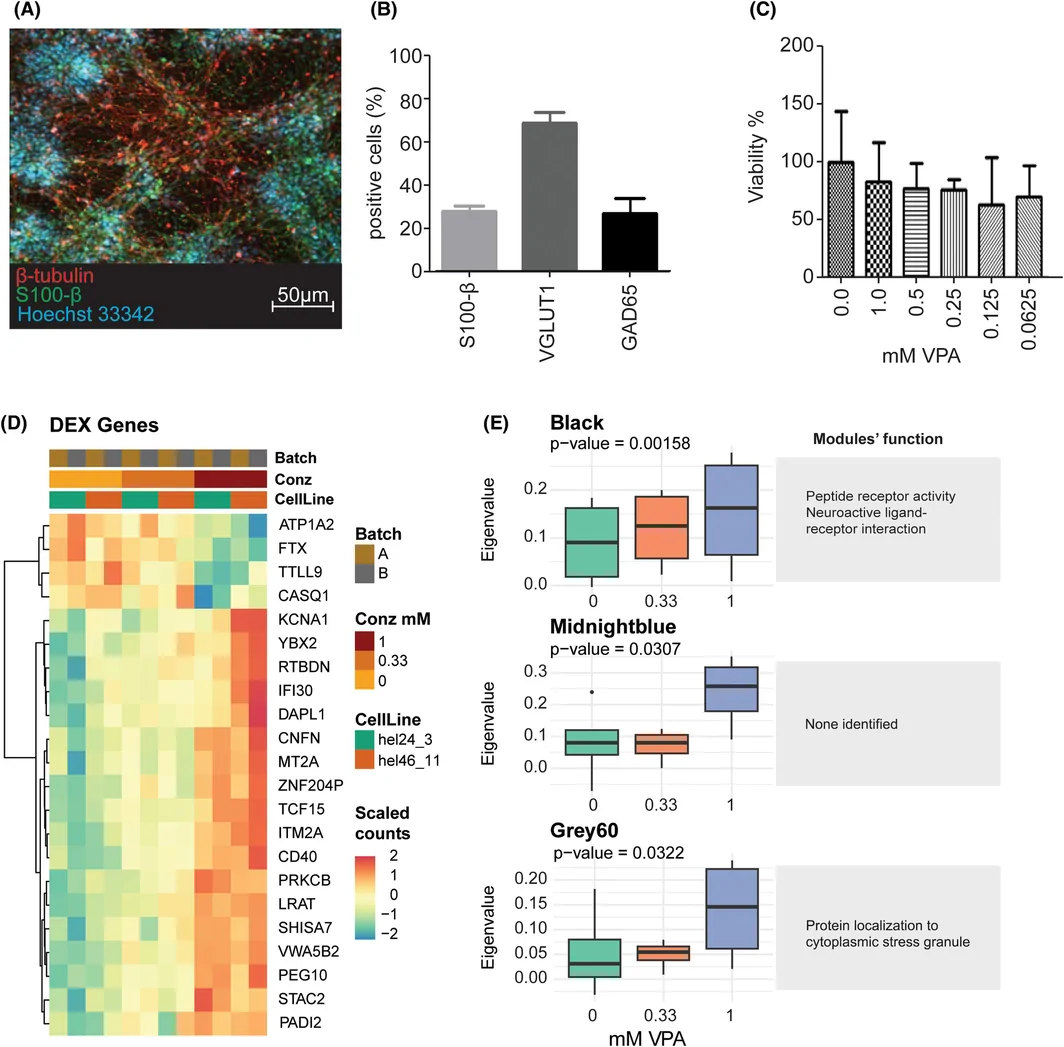
Identification of genes and gene network differentially expressed in induced pluripotent stem cell‐derived neurons: (A) Representative immunohistochemistry (IHC) image of differentiated neurons. Cortical neurons were stained with the neuronal marker 𝛽‐tubulin (red) and glia cells with the S100‐β marker. Nuclei are Hoechst 33342 positive (blue). (B) Quantification of glia cells (S100‐β) as well as glutamatergic (VGLUT1) and γ‐aminobutyric acidergic (GAD65) neurons based on IHC staining. Proportions refer to Hoechst‐stained nuclei. (C) Viability assay determining the optimal concentration of valproic acid (VPA). Because the physiologically reported concentrations of 1 mmol·L^−1^ and .33 mmol·L^−1^ showed acceptable viability, these were chosen for treatment. Percentages refer to the mean viability at 0 mmol·L^−1^. (D) Heatmap of differentially expressed (DEX) genes upon valproate treatment. Only genes with a |log_2_FC| > 1 and false discovery rate (FDR) < .05 were considered. (E) Modules identified in the weighted gene coexpression network analysis with a differential eigengene value expression upon VPA treatment (|log_2_FC| > 1 and FDR < .05). All three modules were upregulated. Results of Gene Ontology term enrichment analysis are displayed below the modules.

iPSC‐derived neurons at D40 expressed β3‐tubulin, VGLUT‐1, GAD65, and S100β, confirming neuronal identity (Figure 2A,B). Cell viability after VPA exposure (Figure 2C) was assessed and clinical concentrations of .33 and 1 mmol·L^−1^ were used. DEX analysis identified 22 genes associated with VPA concentration (Figure 2D), including *ATP1A2* (log_2_FC = −1.836, FDR = .014) and *KCNA1* (log_2_FC = 2.620, FDR = .043), both linked to epilepsy.^31^, ^32^ The coding regions of these genes (±2 kb) and their eQTLs yielded 4093 SNPs used for PBSNP feature engineering.

WGCNA identified three VPA‐upregulated coexpression networks (Figure 2E): (1) Black module: 595 genes (neuroactive ligand‐receptor interaction), (2) Midnightblue module: 269 genes (function unknown), and (3) Gray60 module: 243 genes (localization to cytoplasmic stress granule). These networks showed moderate stability with adequate preservation. Although exploratory in nature, they here formed the basis for PBSEQ graph feature generation.

### 3.3 Feature engineering and generation

#### 3.3.1 PBSNP AE training results and feature extraction

AEs yielded 410 features (Gene Set 1) and 441 features (Gene Set 2), with low reconstruction error (root mean square error = .217 and .267, respectively). Key architectural differences included bottleneck activation and input dropout (Table S3). PBSNP features showed minimal correlation with population stratification (SNP‐based principal components PC1–PC5; mean *r* = −.010, SD = .094).

#### 3.3.2 PBSEQ graph network feature computation

Exome sequencing identified on average 1120 LGD variants (including rare and common single nucleotide variants [SNVs]) per individual. A total of 36 graph‐based PBSEQ features with interindividual variance above zero were calculated.

#### 3.3.3 Feature selection results

Boruta feature selection identified 22 relevant features: 16 PBSNPs (14 from Gene Set 1, three from Gene Set 2), two PBSEQ metrics (sum connectivity, mean betweenness in Gray60 module), and three clinical features (focal seizures, generalized seizures, structural magnetic resonance imaging findings).

### 3.4 Classifier training and testing

**FIGURE 3:**
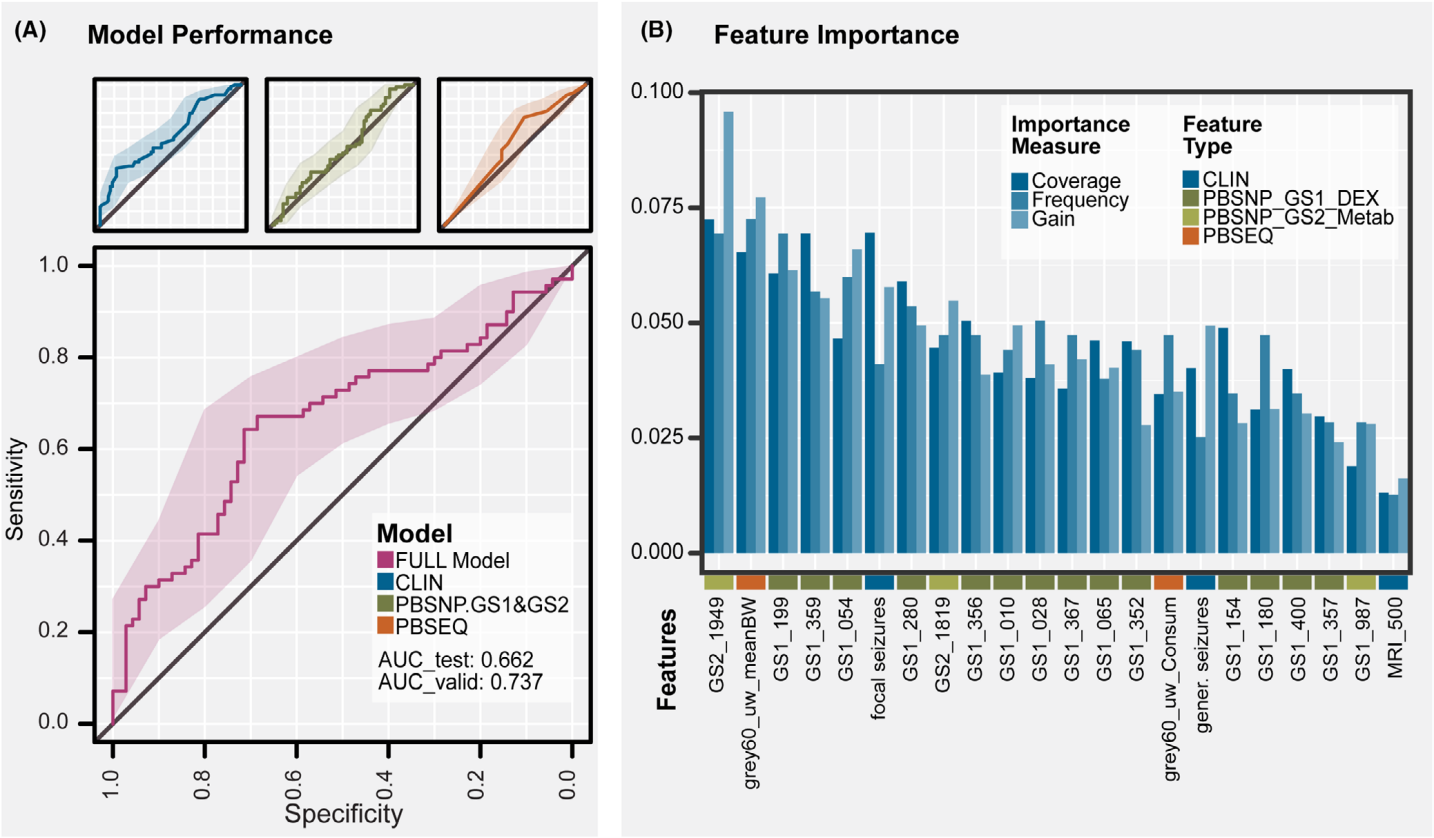
Model performance and feature importance. (A) We compared performance of models including different feature sets. The best model included all three types of features (FULL Model). None of the other models achieved a comparable accuracy when including only clinical data (CLIN), pathway‐based single nucleotide polymorphisms (PBSNP), or pathway‐based sequencing (PBSEQ), respectively. For a full list of performance metrics, see Table S6. AUC_test refers to the unseen test–discovery cohort and AUC_valid to the newly recruited validation cohort. (B) The full model included a total of 22 features, namely, 14 PBSNP features from Gene Set 1 (GS1; differentially expressed [DEX] genes, olive green), three from Gene Set 2 (GS2; Metabolism [Metab] and pharmacodynamics/‐kinetics, light green), two PBSEQ network features (orange), and three clinical phenotypes (blue). Features are sorted based on the average of the three importance measures implemented. AUC, area under the curve. BW, Betweeness; Consum, connectivity sum; DEX, differentially expressed genes; Metab, metabolig genes; gener., generalized; GS, Gene‐set; uw, unweighted;

A gradient‐boosted tree model (XGBoost) was trained on the selected features. After hyperparameter tuning, the best model was trained for 158 iterations and reached a test root mean squared logarithmic error (RMSLE) of .417. In the holdout test set, performance exceeded chance, with AUC = .66 (95% CI = .57–.75), accuracy (ACC) = .66 (95% CI = .58–.74), negative predictive value (NPV) = .66 (95% CI = .54–.76), and positive predictive value (PPV) = .67 (95% CI = .55–.78; Figure 3A, Table S6). Top predictors (Figure 3B) were primarily PBSNP features from Gene Set 1. However, the most important individual features included one from Gene Set 2 and a PBSEQ feature (mean betweenness, Gray60 module).

To assess potential bias, we compared clinical variables between true positives (TP) and false negatives (FN), and between true negatives (TN) and false positives (FP). No significant differences were found in age, sex, or ethnicity, suggesting consistent model performance across demographic groups.

However, focal seizures were significantly less common in TP (26.3%) versus FN (92.0%), and drug resistance was lower in TP (26.3%) versus FN (44.0%; *p* < .001; Table S7). No significant differences were observed between the TN and FP groups.

To assess the contribution of each data modality, six models using features from individual or paired modalities (PBSNP, PBSEQ, CLIN) were compared to the full model. None outperformed the full model in AUC, ACC, sensitivity, specificity, PPV, or NPV. Only the full model consistently performed above chance for all metrics, whereas no other model did for more than one metric (Table S6). From the single‐modality models, CLIN‐only had the highest accuracy (.57, 95% CI = .48–.65) and AUC (.64, 95% CI = .55–.73).

### 3.5 Performance in newly recruited cohort

For the above reasons, the full model was evaluated in the independent validation cohort (Table S5). It achieved robust performance, with a balanced AUC of .73 (95% CI = .63–.83), ACC of .63 (95% CI = .52–.73), NPV of .70 (95% CI = .51–.85), PPV of .60 (95% CI = .46–.72), and sensitivity of .80 (95% CI = .66–.91), indicating adaptability of the threshold to clinical goals.

### 3.6 Avoidable delay estimation

Assuming a 66% overall ASM response rate and published ASM success rates across treatment lines (50.5%, 11.6%, 4.1%)^3^ with a median 162‐day time‐to‐failure per trial,^6^ current trial‐and‐error strategies result in an average delay of 32.1 days before reaching an effective treatment. Using our model with a 60% PPV (validation cohort) reduces this delay to 12.7 days, a 19.4‐day reduction (60.1%). This corresponds to the number needed to treat of approximately 11 (absolute response increase = 9.5%), meaning that one additional patient would benefit for every 11 treated using the model rather than standard first‐line selection.

## 4 DISCUSSION

We developed and validated a machine learning‐based decision‐support tool to predict VPA treatment response across diverse epilepsy types. By integrating features derived from genetic and transcriptomic as well as clinical data, the model outperformed clinically guided trial‐and‐error methods in representative cohorts.^4^, ^33^ This is notable given that success rates typically decline with each subsequent ASM trial, and only ~66% of patients achieve seizure freedom with appropriate therapy.^5^ Our model offers a one‐step prediction strategy comparable to the effectiveness of up to three sequential ASM trials.

### 4.1 Suitability and scalability of the algorithm

The model showed high sensitivity (.80) in the independent validation cohort, supporting its use as a screening tool to identify patients likely to benefit from VPA. Although specificity was lower, prioritizing sensitivity is clinically reasonable to avoid missing potential responders. In this context, model‐guided selection could reduce time to effective treatment by approximately 60%, based on the observed PPV of .60 in the balanced validation cohort.

Practical implementation depends on the availability, cost, and turnaround time of genetic data. Whereas SNP arrays are inexpensive (<€30) and rapidly processed, WES remains costlier (~€1000), with turnaround times ranging from days to weeks. The estimated time gain (mean ~ 19 days, substantially higher in some cases due to long ISC observation windows^4^, ^6^) suggests feasibility if genetic data are available early or can be generated within a clinically relevant timeframe. Importantly, the incremental predictive value of each modality must be weighed against these resource requirements, and formal health economic evaluations are warranted, weighing the cost–benefit ratio between a full model and, for example, the PBSNP+CLIN model.

The model was developed in observational cohorts, where treatment allocation reflects clinical judgment. Accordingly, it is not intended to estimate causal treatment effects but to support decision‐making within real‐world prescribing contexts. Importantly, treatment discontinuation due to adverse effects was included in the definition of nonresponse. Model predictions therefore reflect overall treatment success in clinical practice, implicitly integrating both efficacy and tolerability. In this context, the continued clinical relevance of VPA—despite more restrictive use—supports its selection as a target for prediction, particularly in scenarios requiring careful benefit–risk assessment.

Inclusion of patients with focal epilepsy introduces heterogeneity, as this group is often more treatment‐refractory and VPA may be used later in the disease course. However, the model aims to support decisions across heterogeneous real‐world populations rather than highly selected subgroups. In stratified analyses, focal epilepsy cases were predominantly classified as nonresponders, consistent with clinical expectations, suggesting that the model captures clinically meaningful patterns. Nevertheless, subtype‐specific models may further improve performance in more homogeneous cohorts.

Both cohorts included substantial proportions of nonresponders (53% in discovery, 71% in validation). Treatment line was not included as a predictor, to avoid conflating treatment stage with outcome. The comparable performance across cohorts suggests robustness to these differences, although residual confounding cannot be excluded.

Transcriptomic profiling was performed in neurons derived from healthy donors. Although patient‐derived models may capture disease‐specific responses,^34^ the use of a controlled system enables identification of core pharmacodynamic effects of VPA across heterogeneous epilepsy syndromes. The resulting gene networks are therefore interpreted as generalizable drug‐response mechanisms, although future studies using patient‐derived neurons will be important to refine these findings.

From a technical perspective, the model combines scalable approaches, including AE‐based feature reduction and gradient‐boosted trees, and leverages public resources such as 1000 Genomes and GTEx. Gene set‐based (PBSNP) and network‐derived (PBSEQ) features improved prediction beyond clinical data, supporting their broader applicability. However, the feature learning and transfer approach reduces interpretability and requires SNP overlap with reference panels, potentially excluding relevant variants.

Finally, the cohorts were predominantly of European ancestry, and generalizability to other populations requires further evaluation. Reassuringly, post hoc analyses did not indicate systematic associations between prediction errors and genetic population structure, although validation in more diverse cohorts remains essential.

### 4.2 Comparison to similar approaches

Prior studies using clinical or genetic predictors for ASM response often relied on single‐variable models or small datasets, limiting generalizability. One multimodal study on brivaracetam^35^ used SNV counts per gene set but did not capture nonlinear allele interactions. A deep learning model using 16 clinical variables^36^ achieved moderate performance (AUC = .52–.65), comparable to our CLIN‐only model, underscoring both the feasibility of clinical prediction and the added value of integrating genetic and transcriptomic data.

## 5 CONCLUSIONS

In summary, our machine learning model provides a scalable tool for predicting VPA response, matching the effectiveness of two to three clinician‐guided ASM trials. Early identification of nonresponders may reduce adverse effects, accelerate seizure control, and improve outcomes, with predictions reflecting overall treatment success encompassing both efficacy and tolerability. Future work will extend the model to other ASMs, incorporate additional omics data, and support clinical implementation via a user‐friendly interface. An important next step will be to evaluate incremental benefit of model‐assisted decision‐making beyond standard clinical evaluation.

## AUTHOR CONTRIBUTIONS

**Simeon Platte:** Data curation; formal analysis; methodology; software; validation; visualization; writing—original draft preparation; writing—review & editing. **Afsheen Kumar:** Data curation; formal analysis; methodology; software; writing—review & editing. **Giorgia Guerini:** Investigation; writing—review & editing. **Massimo Pandolfo:** Investigation; resources; visualization; writing—review & editing. **Denise Haslinger:** Data curation; visualization; writing—review & editing. **Colin B. Josephson:** Data curation; investigation; methodology; writing—review & editing. **Guillermo Delgado‐García:** Investigation; writing—review & editing. **Navprabhjot Kaur:** Investigation; writing—review & editing. **Michaela‐Pauline Lux:** Conceptualization; writing—review & editing. **Heiko Stempfle:** Conceptualization; writing—review & editing. **Chantal Depondt:** Investigation; resources; writing—review & editing. **Reetta Kälviäinen:** Investigation; writing—review & editing. **Felix Rosenow:** Investigation; resources; writing—review & editing. **Sophie von Brauchitsch:** Investigation; writing—review & editing. **Karl Martin Klein:** Conceptualization; data curation; formal analysis; funding acquisition; investigation; methodology; project administration; resources; supervision; writing—review & editing. **Andreas G. Chiocchetti:** Conceptualization; data curation; formal analysis; funding acquisition; methodology; project administration; resources; software; supervision; validation; visualization; writing—original draft preparation; writing—review & editing.

## FUNDING INFORMATION

This work was mainly funded by the ERA PerMed Joint Transnational Call 2018, cofunded by the European Commission and national/regional funding partners (project ID: 101137129;RAISE‐GENIC). Partial funding was provided by European Commission, Horizon 2020 (project AIMS‐2‐TRIALS, no. 777394) and European Commission, Horizon Europe‐funded R2D2‐MH (no. 101057385). This work is part of the Centers for Common Disease Genomics (CCDG) program, funded by the National Human Genome Research Institute (NHGRI) and the National Heart, Lung, and Blood Institute. CCDG‐funded Epi25 research activities at the Broad Institute, including genomic data generation on the Broad Genomics Platform, are supported by NHGRI grant UM1 HG008895 (principal investigators [PIs]: Eric Lander, Stacey Gabriel, Mark Daly, Sekar Kathiresan). The Genome Sequencing Program efforts were also supported by NHGRI grant 5U01HG009088–02. The content is solely the responsibility of the authors and does not necessarily represent the official views of the National Institutes of Health. We thank the Stanley Center for Psychiatric Research at the Broad Institute for supporting the genomic data generation efforts. Supplemental Epi25 phenotyping was supported by “Epi25 Clinical Phenotyping R03,” National Institutes of Health (1R03NS108145–01; PIs: Daniel Lowenstein, Samuel Berkovic).

## CONFLICT OF INTEREST STATEMENT

None of the authors has any conflict of interest to disclose. We confirm that we have read the Journal's position on issues involved in ethical publication and affirm that this report is consistent with those guidelines.

## Supporting information


Figure S1.

Figure S2.

Methods S1.

Table S1.

Table S2.

Table S3.

Table S4.

Table S5.

Table S6.

Table S7.

Table S8.

## Data Availability

All software used is open source (Python 3.10 and in R 4.3.3). The full code is publicly available at https://gitlab.rz.uni‐frankfurt.de/cap_molgenlab/epi‐vpa‐ml‐pipeline. Pretrained models and weights as well as patient data are available upon request, via the Epi25 consortium Memorandum Of Understanding.
